# Associations Between 24-Hour Physical Behavior, Self-Perceived Stress, and Coping Self-Efficacy in Everyday Life: Ambulatory Assessment Study

**DOI:** 10.2196/81502

**Published:** 2026-05-22

**Authors:** Katrin Bonn, Doreen Wohlfarth, Irina Timm, Oliver Bender, Ulrich W Ebner-Priemer, Marco Giurgiu

**Affiliations:** 1 Student Health Management, Baden-Wuerttemberg Cooperative State University Karlsruhe, Erzbergerstraße 121, Karlsruhe, Baden-Wuerttemberg, 76133, Germany, 49 721 9735 615; 2 Mental mHealth Lab, Institute of Sports and Sports Science, Karlsruhe Institute of Technology, Karlsruhe, Baden-Wuerttemberg, Germany; 3 Division of Sports and Rehabilitation Medicine, University Hospital Ulm, Ulm, Baden-Wuerttemberg, Germany; 4 Institute of Movement Therapy and movement-oriented Prevention and Rehabilitation, German Sport University Cologne, Cologne, North Rhine-Westphalia, Germany

**Keywords:** ambulatory assessment, accelerometry, coping, mobile health, mHealth, perceived stress, sleep

## Abstract

**Background:**

Psychological stress poses a risk to mental and physical health and has become a major public health challenge. As physical behaviors (ie, physical activity, sedentary behavior (SB), and sleep) play a key role in mental well-being, their targeted modification could be an approach to coping with stress in everyday life. Previous studies have primarily either analyzed the associations between isolated physical behaviors and stress-related outcomes or employed cross-sectional designs. Accordingly, there is a need for deeper insights into the within- and between-person associations between physical behavior over a 24-hour cycle and psychological stress in naturalistic settings.

**Objective:**

This study aimed to investigate how 24-hour physical behavior compositions are associated with daily self-perceived stress and stress-related coping self-efficacy and how replacing time in one behavior with another is linked to changes in both stress-related indicators.

**Methods:**

A total of 198 healthy university employees (mean age 35.87 y, SD 10.76; 109, 54.8% female) participated in a 15-day ambulatory assessment study. Participants reported their momentary stress and coping self-efficacy perceptions up to 6 times a day in electronic diaries. 24–hour physical behavior was measured using a thigh-worn Move 4 accelerometer. The movement data were then classified on a daily basis into the 4 behavior categories of sleep, SB, light physical activity (LPA), and moderate-to-vigorous physical activity (MVPA). In order to obtain physical behavior compositions, an isometric log-ratio transformation was applied, resulting in 4 different sets. The associations between physical behavior compositions, self-perceived stress, and coping self-efficacy were analyzed using 2-level mixed multilevel models. Exploratory reallocation models were conducted to simulate the effects of time shifts from one behavior to another on both stress-related outcomes.

**Results:**

The geometric average day comprised 33.9% (8.1/24 h) sleep, 45.2% (10.8/24 h) SB, 15.8% (3.8/24 h) LPA, and 5.1% (1.2/24 h) in MVPA. More time spent sleeping compared to being sedentary was associated with lower self-perceived stress (standardized *β*=–.03; *t*_1988_*=*–2.045; *P=*.04) but not with coping self-efficacy (*t*_1988_=–1.333; *P=*.18) in the 24-hour cycle. The ratio of SB to the other physical behaviors and time spent in LPA or MVPA relative to SB showed no association with either stress-related outcome. Significant random effects indicate high individual variability in the analyzed associations. The exploratory substitution of SB by LPA, MVPA, or sleep showed no significant changes in self-perceived stress or coping self-efficacy within a 60-minute period.

**Conclusions:**

Adapting 24-hour physical behavior seems to be a promising approach to reduce stress in everyday life, for example, by extending sleep duration instead of being awake in SB. Further research should be conducted on contextual and personal influencing factors in order to develop tailored stress management interventions for the 24-hour cycle.

## Introduction

The feeling of stress is a familiar human experience in everyday life. In particular, high, recurrent, and long-lasting psychological stress is thought to be associated with various physical and mental illnesses, such as coronary artery disease or depression [1-4]. Furthermore, a link is suggested between higher stress levels and an increased risk of mortality [5,6]. Across several studies, reviews indicate that the prevalence of self-reported stress during the COVID-19 pandemic ranged from 32% to 56%, with a focus on both the general population and specific subgroups, such as patients, health care workers, pregnant women, and students [7-9]. Among higher education employees from 16 different countries, 73% reported experiencing moderate to high levels of stress [10]. Psychological stress has become a public health issue with far-reaching implications for society [11], such as an increased risk of substance misuse or addiction [12-14], poorer relationship quality [15-17], or the financial burden resulting from stress-related productivity losses and absenteeism [11,18].

Studies indicate that coping self-efficacy, which refers to the belief that one is capable of effectively managing one’s own stress [19-21], might contribute to effective coping [22] and reduced perceived stress in different target groups [23-25]. Numerous studies have explored associations between individual physical behaviors, including physical activity (PA), sedentary behavior (SB), or sleep, with psychological stress and coping strategies. PA was found to exhibit a medium beneficial effect on stress in adults [26], although the association is characterized by a high degree of individual variability [27,28]. More inconclusive results are reported in studies on the association between indicators of SB and stress [29-33]. Thus, further studies are required that take different time frames, contexts, and domains into consideration [27,30,33]. An insufficient sleep duration appears to be another indicator of experiencing more stress [34-36], while sleep quality was positively associated with higher coping self-efficacy in adolescents [23]. Since physical behaviors do not occur in isolation, their interrelationships must be taken into account. This is addressed by the growing paradigm in sport and health science, which conceptualizes physical behaviors as compositional elements within a 24-hour cycle [37,38]. Reflecting this perspective, recent practice-oriented activity guidelines—such as those promoted under the motto “Make Your Whole Day Matter”—increasingly emphasize the need for balanced time allocation across PA, SB, and sleep, including the recommendation to limit screen time [39,40].

A growing body of literature addresses physical behavior over a 24-hour period and its associations with mental health. Current systematic reviews highlight the positive effects of replacing time in SB with PA for mental health in adults and children [41-43]. For example, among adults, spending 15 minutes more in light physical activity (LPA) instead of being sedentary was associated with lower anxiety, depression, and stress scores [44]. Additionally, reallocating 30 minutes in SB to moderate PA in women or to walking in men aged 21 to 35 years both resulted in a decline in self-reported stress levels [45]. Other cross-sectional studies indicate that replacing time spent in SB with sleep in adults is also associated with lower perceived stress and improved mood [46,47]. However, we are not aware of any study that has examined the association between 24-hour physical behavior and stress-related coping. Overall, much of the evidence linking physical behavior to perceived stress is derived from cross-sectional designs [41,45,47] that analyze between-person differences at a single time point and are therefore inherently limited in their ability to elucidate temporal within-person dynamics, including day-to-day variability in 24-hour physical behavior and stress [48,49].

To overcome these limitations, intensive longitudinal studies (also known as ambulatory assessment or ecological momentary assessment) are currently the state‐of‐the‐art methodology for examining within‐person associations [50,51]. These study designs have several advantages, namely, the assessment in everyday life, in real‐time, with device‐based methods, and repeated measurements with a high sampling frequency, which enables researchers to track dynamic relationships [48,52]. Therefore, they bypass laboratory distortions and minimize recall biases associated with traditional approaches [53]. Accordingly, the implementation of ambulatory assessments is promising for capturing fluctuations in stress-related processes and their associations with physical behavior throughout the day [54]. To the best of our knowledge, no previous study has focused on this within-person interplay. We aimed to investigate the association of device-based 24-hour physical behavior, self-perceived stress, and coping self-efficacy in everyday life. Based on previous empirical findings and theoretical models [42,46], we expected that the composition of 24-hour physical behavior is associated with both daily perceived stress (hypotheses 1‐4) and coping self-efficacy (hypotheses 5‐8) in healthy adults.

We hypothesized that the ratio of SB to sleep, LPA, and moderate-to-vigorous physical activity (MVPA) would be positively associated with self-perceived daily stress (H1), whereas more time spent in (1) MVPA relative to SB (H2), (2) LPA relative to SB (H3), and (3) sleep relative to SB (H4) would be associated with less self-perceived stress. Furthermore, we expected the proportion of SB relative to the other physical behaviors to be negatively associated with coping self-efficacy (H5), while a higher ratio of (1) MVPA to SB (H6), (2) LPA to SB (H7), and (3) sleep to SB (H8) is associated with a stronger belief in being able to cope with tasks at hand. Additional exploratory analyses were conducted to examine the effects of reallocating time from one physical behavior to another on perceived stress and coping self-efficacy. Furthermore, subjective sleep quality and exercise behavior were exploratively fitted into the existing models in order to obtain indications of the robustness of results.

## Methods

### Ethical Considerations

This study was approved by the Ethics Committee of the Karlsruhe Institute of Technology (A2020-001). Prior to the start of the study, participants received comprehensive information about the aim and procedure of the investigation and provided informed consent. Participants had the option of withdrawing from the study at any time. In order to protect participants’ privacy and confidentiality, we deidentified the data after collection. The successful completion of the study was rewarded with a fitness tracker worth €100 (US $114.24).

### Participants

Potential participants were recruited via flyers, mailings, word of mouth, and an internal newsletter from the Karlsruhe Institute of Technology. The study leaders consulted with interested parties via email or telephone to ensure that all predefined inclusion criteria for the study were met. Eligibility criteria included predominantly sedentary work and the absence of current physical injuries or psychological disorders. In total, 222 university employees participated in the study between October 2022 and December 2023 in Karlsruhe, Germany.

### Study Design

This study represents an intensive longitudinal method [55] and is a secondary analysis of a within-person encouragement design investigation, the details of which can be found in Giurgiu et al [56]. However, no overlapping analyses were conducted.

The study started with an in-person session during which participants received detailed information about the study procedures, completed a questionnaire assessing demographics, and were equipped with a study smartphone (Nokia G50 or 6; Nokia Corporation), a Fitbit wearable (Fitbit Inspire 2; Google), and a Move 4 accelerometer (Movisens GmbH). The ambulatory assessment period began the following day and lasted 15 working days. To maintain motivation and ensure compliance, weekend days were not included [57]. During the study period, participants were asked to wear the Fitbit wearable on their wrist and the Move 4 sensor on their thigh as continuously as possible. Moreover, the smartphone prompted participants up to 8 times per day to answer short e-diary questionnaires, with e-diaries occurring based on a wearable-triggered sedentary algorithm, except for the additional morning and evening queries.

### Measures

#### 24-Hour Physical Behavior

Triaxial accelerometer data assessed by a Move 4 sensor (Movisens GmbH) with a sampling frequency of 64 Hz and a range of ±16 gravitational acceleration were used to measure 24-hour physical behavior. The raw accelerometer data were preprocessed after the study period with the software ActiPASS (version 2024.05; ActiPASS) [58]. ActiPASS implements algorithms for nonwear, sleep detection, posture, and activity intensity derived from cadence measures that have demonstrated high accuracy for wake-time movement behaviors and sleep in validity studies [59-61]. Based on the preprocessed data, we aggregated the most commonly examined physical behavior categories—sleep, SB (sitting or lying episodes outside of sleep intervals), LPA (walking with a cadence <100 steps/min and standing), and MVPA (walking with a cadence ≥100 steps/min)—as average minutes per day [38]. In line with previous wearing protocols [62], participants and days were only included when (1) participants had at least 3 valid wear-time days (≥20 h of wear time per day), (2) at least 1 period of walking was detected that day, and (3) days comprised more than zero minutes of sleep.

#### Momentary Self-Perceived Stress and Coping Self-Efficacy

Participants received all e-diaries on the study smartphone (Nokia G50 or 6). A morning and an evening survey were conducted between fixed time periods (morning: 7 AM-8:30 AM, evening: 9:45 PM-11 PM), during which participants were permitted to respond at their chosen time. The e-diary assessments during the day occurred up to 6 times per day between 8:40 AM and 9:40 PM. They were based on a sedentary-triggered design [63], appearing after 30 consecutive minutes in a sitting or lying body position. While half of the triggers exclusively prompted questions on momentary constructs such as affective states or perceived stress, the other half included a prompt to interrupt SB for 3 minutes before answering the identical e-diary questions. To reduce participants’ burden, prompts could be delayed up to 15 minutes and did not occur within at least 30 minutes after a completed e-diary rating.

Momentary perceived stress and coping self-efficacy were each captured by one item in the e-diary assessments completed during the day and then aggregated at the daily level. Previous studies showed single-item measures to be valid and reliable for stress measurement [64]. Both items were based on the Perceived Stress Scale-10 [65]. Following Jones et al [31], participants were asked to rate their subjectively perceived stress level on a visual Likert scale from 1 to 5 (“Do you feel nervous or stressed at the moment?,” 1=“not at all” to 5=“extremely”). Based on the question “Do you currently feel that you can cope with all your upcoming tasks?,” we assessed participants’ current coping self-efficacy on a Likert scale ranging from 1=“not at all” to 5=“extremely.”

#### Covariates

In the initial in-person session, age in years as well as sex (female vs male) were assessed through self-reports, and anthropometric data were objectively measured in order to calculate the BMI. One adapted item from the Pittsburgh Sleep Quality Index [66] was implemented in the morning survey to assess the perceived sleep quality of the previous night on a scale from zero to 100 (“How would you rate your sleep quality last night?”). In the evening survey, participants used a dichotomous response format to indicate whether they had exercised that day (yes vs no). A definition of the term “sports exercise” was not provided.

### Statistical Analysis

Prior to compositional data analysis procedures, we used an isometric log-ratio (ilr) transformation to account for the multicollinearity of direct composition measures [67]. The compositions of sleep, SB, LPA, and MVPA were expressed as ilr coordinates, containing information about one activity in relation to the remaining physical behaviors in the 24-hour cycle. In accordance with existing literature, 3 zero values were replaced before the transformation using the log-ratio expectation maximization algorithm [68].

As is the state of the art when analyzing hierarchically structured intensive longitudinal data, we used a multilevel approach [69]. Prior to fitting the multilevel models, we determined intraclass correlations (ICCs) using unconditional models with daily averaged self-perceived stress (hypotheses 1‐4) and coping self-efficacy (hypotheses 5‐8) as outcomes. Afterwards, multilevel models with repeated measurements (level 1) nested within participants (level 2) were estimated using restricted maximum likelihood and an unstructured covariance matrix for the random effects. In the interest of model parsimony, nonsignificant random effects were excluded. Model assumptions, including multicollinearity, homoscedasticity, and normal distribution of residuals, were checked [69,70]. The covariates *age* (years), *sex* (female vs male*), BMI* (kg/m^2^), and *coping self-efficacy* (only in hypotheses 1-4) were selected a priori and added as predictors based on data availability as well as known associations [71-79]. The respective compositions represented by ilr1, ilr2, and ilr3 were centered within clusters in advance and integrated as predictors. The following equation model refers to hypothesis 1 (further models are presented in Supplementary File A in Multimedia Appendix 1).

H1: *Y* (stress perception)*_ij_* = *γ*_00_ + *γ*_01_ × *sex_j_* + *γ*_02_ × *age_j_* + *γ*_03_ × *BMI_j_* + *γ*_10_ × *coping self-efficacy_ij_* + *γ*_20_ × ilr1 (SB/LPA×MVPA×Sleep)*_ij_* + *γ*_30_ × ilr2 (LPA/MVPA×Sleep)*_ij_* + *γ*_40_ × ilr3 (MVPA/Sleep)*_ij_* + *u*_0_*_j_* + *u*_1_*_j_* × ilr3 (MVPA/Sleep)*_ij_* + *r_ij_*
ilr1 = √(3/4) ln (SB / ∛(LPA×MVPA×Sleep))
ilr2 = √(2/3) ln (LPA/√(MVPA×Sleep))
ilr3 = √(1/2) ln (MVPA/Sleep)

Exploratively, we used substitution analyses to examine the expected change in the outcome when time was reallocated between physical behaviors in a 60-minute window. Pairwise reallocations were calculated in 20-minute increments, with estimates based on the average composition of the included participants [80].

All analyses were performed in R (version 4.4.0; The R Project for Statistical Computing), using the packages *compositions* [81], *zCompositions* [82], and *lme4* [83], with a predetermined *α* level of .05. To facilitate the comparison of results, standardized *β* coefficients were calculated based on established procedures [70].

## Results

### Descriptive Statistics

**Figure 1.**
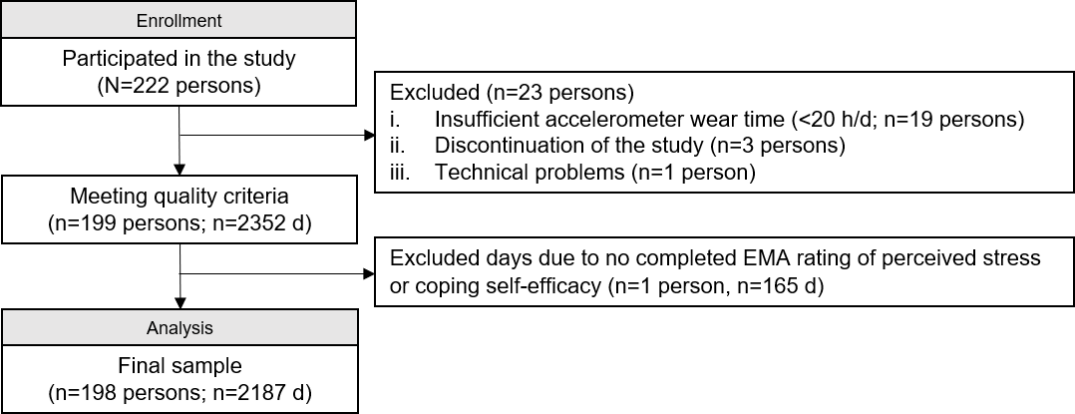
Participant flow chart. EMA: ecological momentary assessment.

Due to insufficient accelerometer wear time (<20 h/d, n=19), discontinuation of the study (n=3), and technical problems (n=1), 23 participants were excluded from the analysis (Figure 1). In total, 199 participants answered momentary assessment prompts on 2352 days, representing an average compliance rate of 69.9% at the personal level. On average, 4.4 surveys were completed per day. Days without any stress or coping self-efficacy rating (n=165 d) were excluded from the analysis, which led to the exclusion of one further participant due to the lack of valid days (n=1). A total of 198 participants were included in the final sample, of whom 109 (54.8%) were female participants, and the average age was 35.87 (SD 10.76) years (Table 1).

**Table 1.** Descriptive characteristics of study participants (N=198, female 54.8%, 109/199).

Variable	Mean (SD)	Minimum	Maximum
Age (y)^a^	35.87 (10.76)	20.18	64.74
BMI (kg/m²)^a^	23.99 (3.28)	17.87	35.25
Valid days^b^	11.84 (3.86)	3	22
Sleep (h/d)^c^	7.95 (1.12)	4.27	12.70
SB^d^ (h/d)^c^	10.51 (1.11)	6.68	14.14
LPA^e^ (h/d)^c^	3.94 (1.00)	1.91	7.33
MVPA^f^ (h/d)^c^	1.35 (0.42)	0.51	2.89
Self-perceived stress^c^	2.34 (0.65)	1.00	4.30
Coping self-efficacy^c^	3.64 (0.65)	1.00	5.00

a Aggregated within participants.

b Accelerometer wear time ≥20 h/d, aggregated within participants.

c Accelerometer wear time ≥20 h/d, aggregated within days and participants.

d SB: sedentary behavior.

e LPA: light physical activity.

f MVPA: moderate-to-vigorous physical activity.

Over the course of all days, participants wore the accelerometer for an average of 23.79 (SD 0.65) hours per day. The 24-hour geometric mean composition of physical behavior for all analyzed days consisted of 33.9% sleep (8.1/24 h), 45.2% SB (10.8/24 h), 15.8% LPA (3.8/24 h), and 5.1% MVPA (1.2/24 h). Further information on participants’ physical behavior, self-perceived stress, and coping self-efficacy is provided in Table 1.

Across all days, the average daily perceived stress score was 2.36 (SD 0.87, mean of per-person minimum=1.49, mean of per-person maximum=3.28), with lower values indicating lower stress perception than higher values (scale: 1‐5). ICC calculations (ρI=0.483) revealed that 51.7% of the variance of the stress perception was based on the within-person fluctuations. The average coping self-efficacy score (scale: 1‐5) was 3.62 (SD 0.85, mean of per-person minimum=2.83, mean of per-person maximum=4.33), where 40.8% of the variance can be explained by within-person variances (ρI=0.592).

### Association Between 24-Hour Physical Behavior and Self-Perceived Stress

Daily self-perceived stress was not significantly associated with time spent in (1) SB relative to the other behaviors (H1), (2) MVPA relative to SB (H2), or (3) LPA relative to SB (H3) (Table 2). In line with hypothesis 4, results indicate a significant negative association between ilr3 (sleep/SB) and self-perceived stress (standardized *β*=–.03; *t*_1988_=–2.045; *P*=.04). Coping self-efficacy and age of the participants were both significantly negatively related to perceived stress in all models (Table 2). The presence of significant random effects in models 1, 2, and 3 indicates that the effects of the ratio of MVPA to sleep, sleep to MVPA and SB, as well as sleep to MVPA, LPA, and SB, vary between individuals (Supplementary File B in Multimedia Appendix 2).

**Table 2.** Multilevel models analyses to predict daily self-perceived stress: random and fixed effects.

Variable	Self-perceived stress											
	H1^a^			H2^b^			H3^c^			H4^d^		
	*B* (SE)^e^/SD (VE)^f^,	95% CI	*P* value	*B* (SE)^e^/SD (VE)	95% CI	*P* value	*B* (SE)^e^/SD (VE)	95% CI	*P* value	*B* (SE)^e^/SD (VE)	95% CI	*P* value
Fixed effects^g^												
Intercept, *ß*_00_	2.29 (0.35)	1.59 to 2.98	<.001	2.26 (0.36)	1.57 to 2.96	<.001	2.26 (0.35)	1.57 to 2.96	<.001	2.26 (0.36)	1.56 to 2.95	<.001
Sex^h^, *ß*_01_	–0.10 (0.09)	–0.28 to 0.07	.26	–0.10 (0.09)	–0.28 to 0.08	.28	–0.10 (0.09)	–0.28 to 0.08	.28	–0.10 (0.09)	–0.28 to 0.08	.27
Age (y), *ß*_02_	–0.01 (0.00)	–0.02 to 0.00	.03	–0.01 (0.00)	–0.02 to 0.00	.04	–0.01 (0.00)	–0.02 to 0.00	.04	–0.01 (0.00)	–0.02 to 0.00	.04
BMI (kg/m^2^), *ß*_03_	0.02 (0.01)	–0.01 to 0.05	.19	0.02 (0.01)	–0.01 to 0.05	.18	0.02 (0.01)	–0.01 to 0.05	.18	0.02 (0.01)	–0.01 to 0.05	.17
Coping self-efficacy, *ß*_10_	–0.57 (0.02)	–0.61 to –0.53	<.001	–0.57 (0.02)	–0.61 to –0.53	<.001	–0.57 (0.02)	–0.61 to –0.53	<.001	–0.57 (0.02)	–0.61 to –0.52	<.001
ilr1^i^, *ß*_20_	0.05 (0.05)	–0.04 to 0.14	.23	–0.02 (0.04)	–0.10 to 0.05	.53	–0.06 (0.06)	–0.17 to 0.05	.28	0.03 (0.03)	–0.03 to 0.09	.33
ilr2, *ß*_30_	–0.01 (0.04)	–0.09 to 0.07	.87	–0.08 (0.06)	–0.19 to 0.03	.14	0.01 (0.04)	–0.06 to 0.08	.78	0.00 (0.03)	–0.06 to 0.07	.94
ilr3, *ß*_40_	0.06 (0.04)	–0.02 to 0.15	.12	–0.02 (0.04)	–0.09 to 0.06	.65	–0.05 (0.04)	–0.12 to 0.03	.22	–0.10 (0.05)	–0.20 to 0.00	.04
Random effects^g^												
Intercept, *u*_0_	0.61 (0.38)	0.55 to 0.68	<.001	0.61 (0.38)	0.55 to 0.68	<.001	0.61 (0.38)	0.55 to 0.68	<.001	0.61 (0.38)	0.55 to 0.68	<.001
ilr3, *u*_1_	0.22 (0.05)	0.13 to 0.31	<.001	N/A^j^	N/A	N/A	N/A	N/A	N/A	N/A	N/A	N/A
ilr2, *u*_1_	N/A	N/A	N/A	0.31 (0.09)	0.18 to 0.43	<.001	N/A	N/A	N/A	N/A	N/A	N/A
ilr1, *u*_1_	N/A	N/A	N/A	N/A	N/A	N/A	0.31 (0.10)	0.19 to 0.44	<.001	N/A	N/A	N/A
Int.-Slope corr.^k^	–0.23	–0.56 to 0.12	N/A	0.09	–0.25 to 0.42	N/A	0.14	–0.19 to 0.46	N/A	N/A	N/A	N/A
Residual, *r*^l^	0.54 (0.29)	0.52 to 0.56	N/A	0.54 (0.29)	0.52 to 0.55	N/A	0.54 (0.29)	0.52 to 0.55	N/A	0.55 (0.30)	0.53 to 0.56	N/A

a Hypothesis 1: ilr1 (sedentary behavior [SB]/(light physical activity [LPA]*moderate-to-vigorous physical activity [MVPA]*Sleep)), ilr2 (LPA/(MVPA*Sleep)), ilr3 (MVPA/Sleep).

b Hypothesis 2: ilr1 (LPA/(Sleep×MVPA×SB)), ilr2 (Sleep/(MVPA×SB)), ilr3 (MVPA/SB).

c Hypothesis 3: ilr1 (Sleep/(MVPA×LPA×SB)), ilr2 (MVPA/(LPA×SB)), ilr3 (LPA/SB).

d Hypothesis 4: ilr1 (MVPA/(LPA×Sleep×SB)), ilr2 (LPA/(Sleep×SB)), ilr3 (Sleep/SB).

e Unstandardized estimates and SEs.

f VE: variance estimate.

g Fixed effects are presented as B (SE); random effects are presented as SD (VE).

h Compared to female.

i ilr: isometric log ratio coordinate.

j N/A: not applicable.

k Intercept-slope correlations are presented as correlation coefficients with 95% CIs.

l Residual values are presented as SD (VE).

### Association Between 24-Hour Physical Behavior and Coping Self-Efficacy

Contrary to our hypotheses, the composition of SB to LPA, MVPA, and sleep was not significantly associated with the daily averaged coping self-efficacy score (H5). Furthermore, none of the other expected associations between the compositions of physical behavior and coping self-efficacy (H6, H7, H8) were observed (Table 3). A positive association between coping self-efficacy and age became evident in all 4 models (H5-H8). Additionally, random effects were found for the ratio of LPA to SB, as well as LPA to all other remaining activities (Table 3).

**Table 3.** Multilevel models analyses to predict daily coping self-efficacy: random and fixed effects.

Variable	Coping self-efficacy											
H5^a^			H6^b^			H7^c^			H8^d^		
*B* (SE)^e^/SD (VE)^f^	95% CI	*P* value	*B* (SE)^e^/SD (VE)	95% CI	*P* value	*B* (SE)^e^/SD (VE)	95% CI	*P* value	*B* (SE)^e^/SD (VE)	95% CI	*P* value
Fixed effects^g^												
Intercept, *ß*_00_	3.36 (0.38)	2.61 to 4.11	<.001	3.36 (0.38)	2.61 to 4.11	<.001	3.37 (0.38)	2.62 to 4.12	<.001	3.36 (0.38)	2.61 to 4.11	<.001
Sex^h^, *ß*_01_	0.14 (0.10)	–0.05 to 0.33	.15	0.14 (0.10)	–0.05 to 0.33	.15	0.14 (0.10)	–0.05 to 0.34	.15	0.14 (0.10)	–0.05 to 0.33	.15
Age (y), *ß*_02_	0.01 (0.00)	0.00 to 0.02	.02	0.01 (0.00)	0.00 to 0.02	.02	0.01 (0.00)	0.00 to 0.02	.03	0.01 (0.00)	0.00 to 0.02	.02
BMI (kg/m^2^), *ß*_03_	–0.01 (0.02)	–0.04 to 0.02	.65	–0.01 (0.02)	–0.04 to 0.02	.64	–0.01 (0.02)	–0.04 to 0.02	.64	–0.01 (0.02)	–0.04 to 0.02	.65
ilr1^i^, *ß*_10_	0.01 (0.05)	–0.08 to 0.10	.85	0.09 (0.04)	0.00 to 0.18	.052	–0.10 (0.05)	–0.20 to –0.01	.02	0.03 (0.03)	–0.03 to 0.09	.37
ilr2, *ß*_20_	0.08 (0.04)	0.00 to 0.15	.06	–0.08 (0.05)	–0.17 to 0.01	.10	–0.01 (0.04)	–0.09 to 0.06	.75	0.08 (0.03)	0.01 to 0.15	.02
ilr3, *ß*_30_	0.08 (0.03)	0.01 to 0.15	.02	0.01 (0.04)	–0.06 to 0.09	.74	0.05 (0.04)	–0.04 to 0.14	.26	–0.07 (0.05)	–0.17 to 0.03	.18
Random effects^g^												
Intercept, *u*_0_	0.67 (0.44)	0.59 to 0.73	<.001	0.67 (0.44)	0.59 to 0.74	<.001	0.67 (0.44)	0.59 to 0.74	<.001	0.67 (0.44)	0.59 to 0.73	<.001
ilr1, *u*_1_	N/A^j^	N/A	N/A	0.28 (0.08)	0.15 to 0.39	.005	N/A	N/A	N/A	N/A	N/A	N/A
ilr3, *u*_1_	N/A	N/A	N/A	N/A	N/A	N/A	0.26 (0.07)	0.12 to 0.38	.02	N/A	N/A	N/A
Int.-Slope corr.^k^	N/A	N/A	N/A	–0.03	–0.37 to 0.31	N/A	–0.11	–0.49 to 0.23	N/A	N/A	N/A	N/A
Residual, *r*^l^	0.56 (0.31)	0.54 to 0.58	N/A	0.55 (0.30)	0.53 to 0.57	N/A	0.55 (0.30)	0.53 to 0.57	N/A	0.56 (0.31)	0.54 to 0.58	N/A

a Hypothesis 5: ilr1 (SB/(light physical activity [LPA]×moderate-to-vigorous physical activity [MVPA]×Sleep)), ilr2 (LPA/(MVPA*Sleep)), ilr3 (MVPA/Sleep).

b Hypothesis 6: ilr1 (LPA/(Sleep×MVPA×SB)), ilr2 (Sleep/(MVPA×SB)), ilr3 (MVPA/SB).

c Hypothesis 7: ilr1 (Sleep/(MVPA×LPA×SB)), ilr2 (MVPA/(LPA×SB)), ilr3 (LPA/SB).

d Hypothesis 8: ilr1 (MVPA/(LPA×Sleep×SB)), ilr2 (LPA/(Sleep×SB)), ilr3 (Sleep/SB).

e Unstandardized estimates and SEs.

f VE: variance estimate.

g Fixed effects are presented as B (SE); random effects are presented as SD (VE).

h Compared to female.

i ilr: isometric log ratio coordinate.

j N/A: not applicable.

k Intercept-slope correlations are presented as correlation coefficients with 95% CIs.

l Residual values are presented as SD (VE).

### Exploratory Analyses

**Figure 2.**
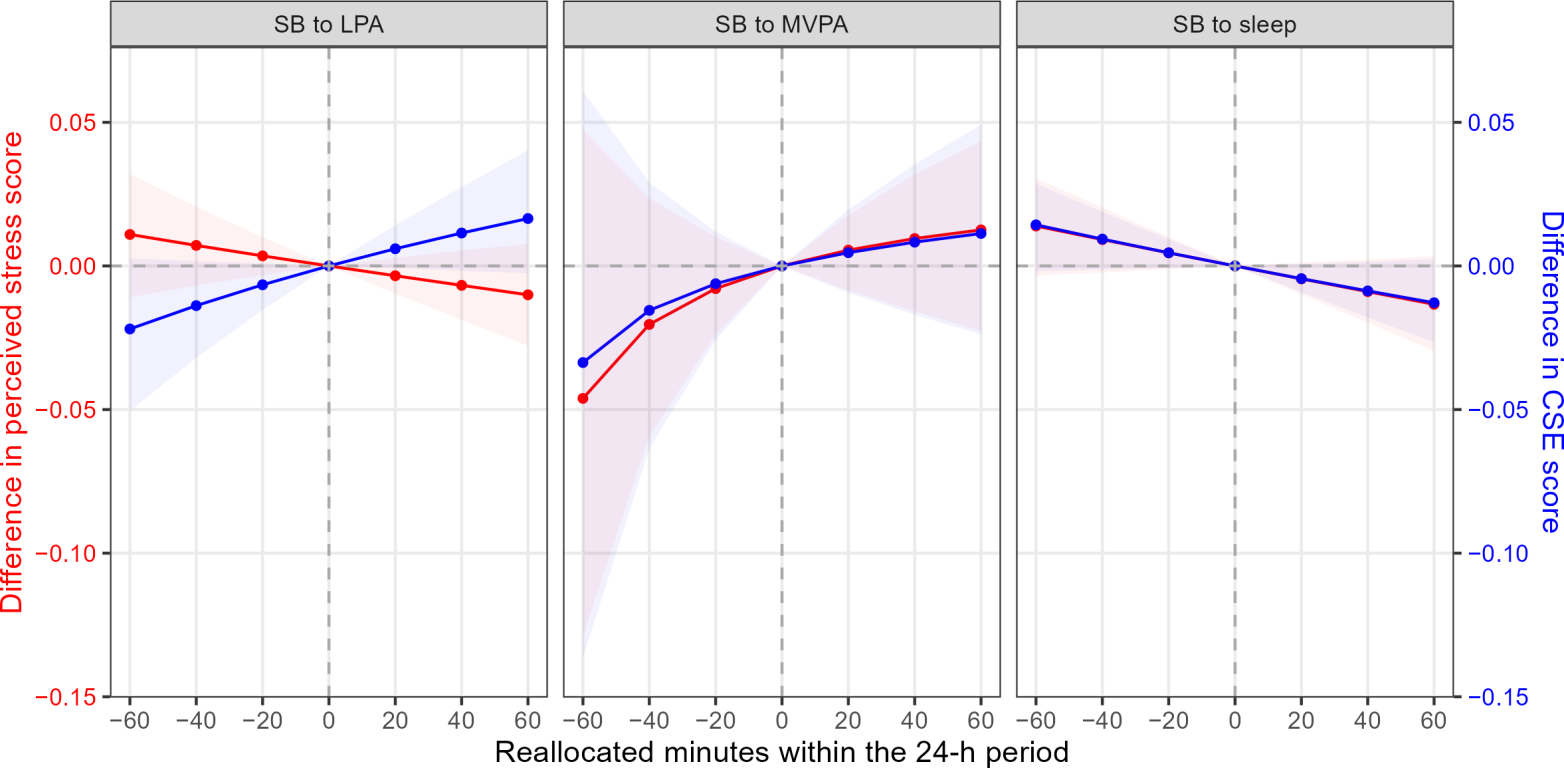
Estimated changes in predicted daily averaged self-perceived stress and coping self-efficacy associated with pairwise reallocations^a^. ^a^95% CIs were derived by bootstrapping with 1000 replicates. CSE: coping-self-efficacy; LPA: light physical activity; MVPA: moderate-to-vigorous physical activity; SB: sedentary behavior.

We exploratively calculated reallocation analyses within a 60-minute frame, simulating the effects of replacing the same amount of time (20-min increments) in one physical behavior with another on self-perceived stress and coping self-efficacy. The starting point represents the empirically observed mean-centered composition of 490 minutes sleep, 649 minutes SB, 227 minutes LPA, and 74 minutes MVPA. The replacement of SB by LPA, MVPA, or sleep was analyzed, with no significant changes of self-perceived stress or coping self-efficacy within a 60-minute period (Figure 2).

As part of our exploratory analyses, self-reported exercise behavior was added as an additional fixed predictor in all models including PA (models 1‐3 and 5‐7; Supplementary File C in Multimedia Appendix 3, Tables1  2). We found no statistically significant association between exercise behavior and perceived stress or coping self-efficacy. However, adding exercise behavior revealed a significant positive association of time in SB relative to the remaining physical behaviors on perceived stress (standardized *β*=.04; *t*_1379_=2.039; *P=*.04). Furthermore, adding subjective sleep quality as a fixed predictor in both models investigating the association of the ratio of sleep to SB revealed sleep quality to be associated with coping self-efficacy (model 8: standardized *β*=.08; *t*_1769_=5.279; *P*<.001) but not with perceived stress (model 4: standardized *β*=–.01; *t*_1769_=–0.724; *P=*.47) (Supplementary File C in Multimedia Appendix 3 and Table 3). The addition of sleep quality did not alter the effect of the ratio of sleep duration to SB on self-perceived stress (standardized *β*=–.03; *t*_1776_=–2.364; *P=*.02).

## Discussion

### Principal Findings

The relationship between individual physical behaviors and stress has been studied extensively in recent years. Our 15-day ambulatory assessment study with 198 participants expands existing evidence with real-time assessments in a naturalistic setting, providing within- and between-subject insights into participants’ everyday lives. We aimed to investigate the associations between device-based 24-hour physical behavior compositions with perceived stress and stress-related coping self-efficacy in an intensive longitudinal data design. Our results indicate that time spent sleeping relative to being sedentary over the 24-hour cycle is negatively associated with perceived daily stress level (H4) but not with coping self-efficacy (H8). The ratios of SB to the remaining behaviors (H1/H5), MVPA to SB (H2/H6), and LPA to SB (H3/H7) were not significantly related to either self-perceived stress or coping self-efficacy. In the exploratory analyses, replacing time spent in SB by LPA, MVPA, or sleep within a 60-minute period did not result in any significant changes in perceived stress or coping self-efficacy.

One possible explanation for why the assumed associations between the composition of SB and the other physical behaviors with perceived stress or coping self-efficacy were not evident could be that the level of mental workload during SB was not taken into account. Research has shown that mentally passive SB, for example, watching television, can increase the risk of depression, while mentally active SB, such as reading, is considered a protective factor [84,85]. As the relationship between SB and stress-related parameters is particularly important for developing targeted health-promoting measures in working environments with high levels of sedentariness, it may be advisable to consider the aggregation of data not on a daily basis but rather within the context of working and nonworking hours [74]. When adding exercise behavior as an additional fixed predictor to our main models (H1/H5), more time in SB compared to the remaining behaviors was associated with higher stress levels. Accordingly, the association between more time in SB compared to the remaining behaviors and stress levels might not be independent of other constructs and influencing factors such as mental load or time of the day. Therefore, future research endeavors might be interested in analyzing the associations between the composition of physical behaviors and momentary stress levels at different aggregation levels (eg, 1 or 2 h) [86].

We found a negative association between the time spent sleeping relative to SB and self-perceived stress, which is in line with previous compositional analysis studies suggesting that replacing sedentary time with sleep is associated with lower psychological stress in young individuals [46] and in working adults [47]. As Bassett et al [87] identified sleep quality, but not sleep duration, to influence cortisol responses to acute psychosocial stress, we exploratively included sleep quality as a further fixed predictor in our model. Sleep quality did not emerge as an additional influencing factor in our model, whereas the association between the ratio of sleep to SB and perceived stress remained significant, underscoring its robustness. Contrary to our hypotheses, a higher ratio of sleep to SB was not related to coping self-efficacy, even after adding sleep quality as a further exploratory fixed effect. The exploratively expanded model revealed a positive association between sleep quality and coping self-efficacy, which is consistent with the findings of Ten Brink et al [23]. This study investigated the importance of coping self-efficacy in breaking the bidirectional cycle between sleep and stress in adolescents. The findings showed that sleep quality not only leads to a higher coping self-efficacy but also a lower level of reported stress the following day [23]. Based on our results, we can only vaguely expect an indirect effect of sleep quality on stress perception, as coping self-efficacy appears to be significantly negatively associated with self-perceived stress in all models (H1-H4). However, these findings emphasize the potential of promoting one’s belief in being able to cope with stress for interventions to reduce stress in everyday life, especially as some areas of work as well as personal obligations do not allow for an increase in sleep duration.

The compositions of LPA to SB and MVPA to SB in the 24-hour cycle were not significantly associated with either perceived stress or coping self-efficacy. In the limited existing literature, Rong et al [45], for example, found that replacing self-reported SB with walking or moderate PA had a positive effect on perceived stress. One reason for the differing results might be that subjective measures, possibly related to common method variance [88], tend to be more predictive of affect-related outcomes than objective measures [89]. Additionally, our study design (ie, half of the assessments were captured after a sedentary bout of ≥30 min) could have resulted in lower daily stress ratings. Our analyses revealed various significant random effects, for example, for the ratio of LPA to SB and LPA to remaining behaviors on coping self-efficacy, thus underlining the variability across participants regarding the association of 24-hour physical behavior and both stress-related outcomes. The occurrence of these within-subject associations should be taken into account in future investigations. Momentary assessments could provide further insights into the temporal sequencing within a day and help assess potential contextual influences, such as the environment of PA, since it has been found that outdoor PA is associated with lower negative affect than indoor PA [90].

Overall, based on our results, we suggest examining the relationship between compositional predictors, psychological stress, and further moderating and mediating factors in more detail. Studies and replications should be conducted with diverse and stress-prone samples, such as students [91], to gain application-oriented insights into the interplay of the 24-hour physical behavior and mental health outcomes. A more comprehensive understanding of the complex association between the mechanisms underlying SB, PA, and sleep and the biological, psychosocial, and behavioral facets of stress could advance a greater focus of the 24-hour approach not only in stress management but also in public health [92,93] and health promotion recommendations [34].

### Limitations

This study has a number of strengths, but a few limitations must be taken into account. The fact that the sample included only university employees with predominantly sedentary working conditions limits the generalizability of the results. Additionally, the observational study design does not allow the elucidation of causalities. Due to its multidimensionality, the assessment of the stress is challenging [94]. In line with previous studies [31,64], we measured both stress-related outcomes with subjective, self-report-based single-item measures that capture only the stress that participants are aware of [95,96]. However, using an ambulatory assessment design promoted the reduction of retrospective biases [50]. Half of our assessments occurred after a sedentary bout, which may have resulted in reduced variance, so that further random sampling studies are required. However, ICC calculations show that both stress-related outcomes can be adequately explained by within-person variance. As we aggregated device-based activity data within days from 12 AM to 11:59 PM, the actual sleep time per day might be distorted, because the time in bed before midnight is not represented as sleep time on the following day. Finally, the implemented sleep algorithm used may incorrectly classify daytime sleep as being sedentary (ie, if not a primary time-in-bed period was detected) [59]. However, this appears to play a subordinate role in the analyses, as daytime sleep was only reported in the evening e-diaries on 5.6% (122/2187) of all analyzed days.

### Conclusions

This study investigated the associations between device-based 24-hour physical behavior, self-perceived stress, and coping self-efficacy in daily life. Our findings suggest that spending more time asleep relative to being sedentary in the 24-hour period is associated with lower levels of perceived stress on the same day. However, the ratio of sleep to SB was not associated with the perceived ability to cope with daily demands. The composition of SB to the other physical behaviors and time in LPA or MVPA relative to SB showed no association with either stress-related outcome. Nonetheless, within-subject associations and descriptive trends of behavioral reallocation hint at the potential relevance of daily activity patterns for handling stressful situations. These findings underscore the need for future ecologically valid studies that incorporate contextual and individual variables, as well as momentary assessments, in order to better understand the role of physical behavior in stress regulation. Such insights are necessary in order to provide target group–appropriate health-promoting recommendations for coping with stress in everyday life.

## Supplementary material

10.2196/81502 Multimedia Appendix 1 Structural equation models.

10.2196/81502 Multimedia Appendix 2 Individual associations of self-perceived stress and the ratio of sleep to moderate-to-vigorous physical activity (MVPA), light physical activity (LPA), and sedentary behavior (SB) at the participant level.

10.2196/81502 Multimedia Appendix 3 Exploratory analyses: results of adapted multilevel models.

## Abbreviations

ICC: intraclass correlation
ilr: isometric log-ratio
LPA: light physical activity
MVPA: moderate-to-vigorous physical activity
PA: physical activity
SB: sedentary behavior
