# Supplementary material for: Associations Between 24-Hour Physical Behavior, Self-Perceived Stress, and Coping Self-Efficacy in Everyday Life: Ambulatory Assessment Study
Source: JMIR Mhealth Uhealth. 2026 May 22;14:e81502. doi: 10.2196/81502 (PMC13197029; doi:10.2196/81502)
Supplement: Multimedia Appendix 1 [file mhealth-v14-e81502-s001.docx]

**Structural equation models**

**Eq. 1: Hypothesis 2**

*H2: Y(stress perception)_ij_ = γ_00_ + γ_01_ * sex_j_ +γ_02_ * age_j_ +γ_03_ * BMI_j_ + γ_10_ * coping self-efficacy_ij_ + γ_20_ * ilr1 (LPA / Sleep* MVPA* SB)_ij_ +γ_30_ * ilr2 (Sleep / MVPA* SB)_ij_ + γ_40_ * ilr3 (MVPA / SB)_ij_ + u0_j_ +u2_j_ * ilr2 (Sleep / MVPA* SB)_ij_ + r_ij_*

**Eq. 2: Hypothesis 3**

*H3: Y(stress perception)_ij_ = γ_00_ + γ_01_ * sex_j_ +γ_02_ * age_j_ +γ_03_ * BMI_j_ + γ_10_ * coping self-efficacy_ij_ +γ_20_ * ilr1(Sleep / MVPA* LPA* SB)_ij_ +γ_30_ * ilr2 (MVPA / LPA* SB)_ij_ +γ_40_ * ilr3 (LPA/ SB)_ij_ + u0_j_ +u2_j_ * ilr1 (Sleep / MVPA* LPA* SB)_ij_ + r_ij_*

**Eq. 3: Hypothesis 4**

*H4: Y(stress perception)_ij_ = γ_00_ + γ_01_ * sex_j_ +γ_02_ * age_j_ +γ_03_ * BMI_j_ + γ_10_ * coping self-efficacy_ij_ + γ2_0_ * ilr1(MVPA / LPA* Sleep* SB)_ij_ +γ_30_ * ilr2 (LPA / Sleep* SB)_ij_ +γ_40_ * ilr3 (Sleep / SB)_ij_ + u0_j_ + r_ij_*

**Eq. 4: Hypothesis 5**

*H5: Y(coping self-efficacy)_ij_ = γ_00_ + γ_01_ * sex_j_ +γ_02_ * age_j_ +γ_03_ * BMI_j_ +γ_10_ * ilr1 (SB / LPA* MVPA* Sleep)_ij_ +γ_20_ * ilr2 (LPA / MVPA* Sleep )_ij_ +γ_30_ * ilr3 (MVPA / Sleep)_ij_ + u0_j_ + r_ij_*

**Eq. 5: Hypothesis 6**

*H6: Y(coping self-efficacy)_ij_ = γ_00_ + γ_01_ * sex_j_ +γ_02_ * age_j_ +γ_03_ * BMI_j_ +γ_10_ * ilr1 (LPA / Sleep* MVPA* SB)_ij_ +γ_20_ * ilr2 (Sleep / MVPA* SB)_ij_ +γ_30_ * ilr3 (MVPA / SB)_ij_ + u0_j_ +u2_j_ * ilr1 (LPA / Sleep* SB* MVPA)_ij_ + r_ij_*

**Eq. 6: Hypothesis 7**

*H7: Y(coping self-efficacy)_ij_ = γ_00_ + γ_01_ * sex_j_ +γ_02_ * age_j_ +γ_03_ * BMI_j_ +γ_10_ * ilr1(Sleep / MVPA* LPA* SB)_ij_ +γ_20_ * ilr2 (MVPA / LPA* SB)_ij_ +γ_30_ * ilr3 (LPA/ SB)_ij_ + u0_j_ +u2_j_ * ilr3 (LPA/ SB)_ij_ + r_ij_*

**Eq. 7: Hypothesis 8**

*H8: Y(coping self-efficacy)_ij_ = γ_00_ + γ_01_ * sex_j_ +γ_02_ * age_j_ +γ_03_ * BMI_j_ +γ_10_ * ilr1(MVPA / LPA* Sleep* SB)_ij_ +γ_20_ * ilr2 (LPA / Sleep * SB)_ij_ +γ_30_ * ilr3 (Sleep / SB)_ij_ + u0_j_ + r_ij_*
