# Supplementary material for: Associations Between 24-Hour Physical Behavior, Self-Perceived Stress, and Coping Self-Efficacy in Everyday Life: Ambulatory Assessment Study
Source: JMIR Mhealth Uhealth. 2026 May 22;14:e81502. doi: 10.2196/81502 (PMC13197029; doi:10.2196/81502)
Supplement: Multimedia Appendix 2 [file mhealth-v14-e81502-s002.docx]

**Individual associations of self-perceived stress and the ratio of sleep to MVPA, LPA and SB at the participant level**

**
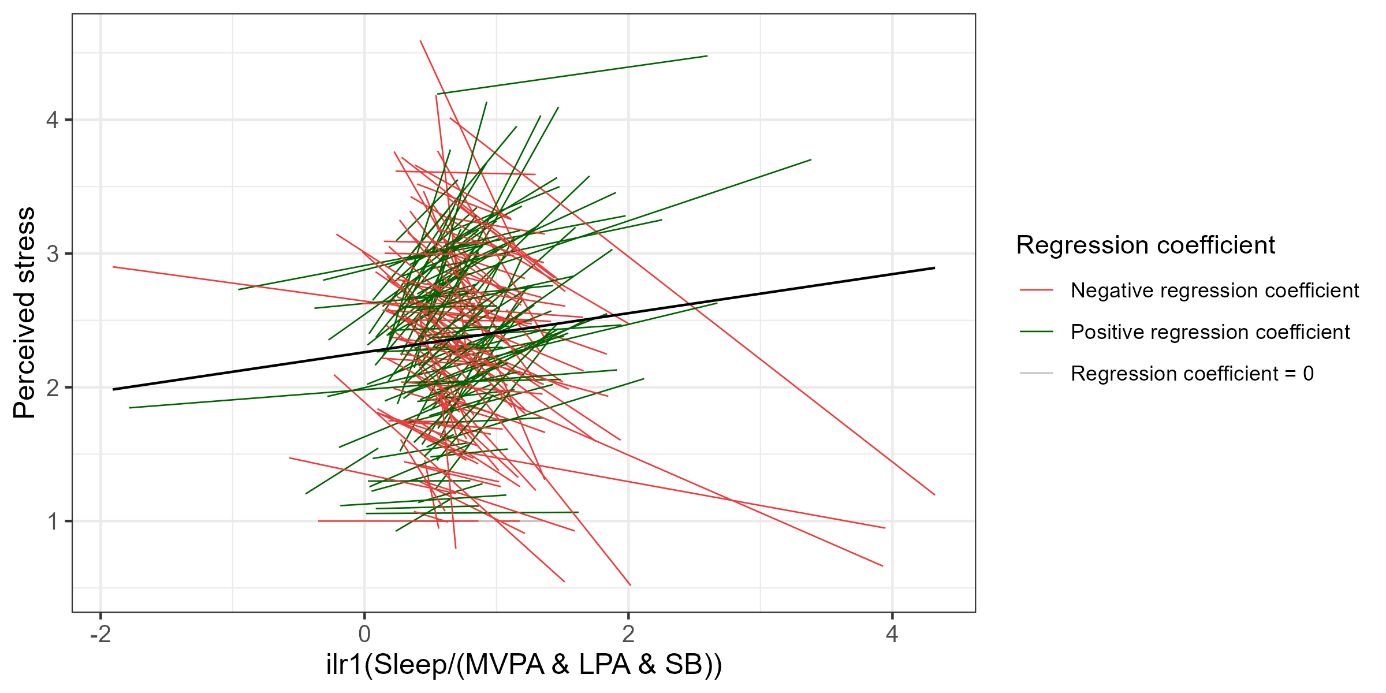
**

**Figure S1.** Spaghetti-Plot showing individual associations between self-perceived stress and not centred ilr1(Sleep/ (MVPA & LPA & SB). Scale of perceived stress ranging from 1 = “not at all” to 5 = “extremely”.

Abbreviations: ilr = isometric log ratio coordinate; LPA = light physical activity; MVPA = moderate-to-vigorous physical activity; SB = sedentary behavior
