# Supplementary material for: Associations Between 24-Hour Physical Behavior, Self-Perceived Stress, and Coping Self-Efficacy in Everyday Life: Ambulatory Assessment Study
Source: JMIR Mhealth Uhealth. 2026 May 22;14:e81502. doi: 10.2196/81502 (PMC13197029; doi:10.2196/81502)
Supplement: Multimedia Appendix 3 [file mhealth-v14-e81502-s003.docx]

**Exploratory analysis: Results of Adapted Multilevel Models**

**Table 1** Adapted multilevel models with daily self-perceived stress as outcome and exercise behavior as additional fixed predictor

|  | H1^a^ | | | H2^b^ | | | | | H3^c^ | | |
| --- | --- | --- | --- | --- | --- | --- | --- | --- | --- | --- | --- |
|  | b (SE)^e^ | 95% CI | *P* value | | b (SE)^e^ | 95% CI | *P* value | b (SE)^e^ | | 95% CI | *P* value |
| **Fixed effects**  Intercept, *_ß00_* | 2.32 (0.37) | [1.60, 3.04] | <.001 | | 2.29 (0.37) | [1.57, 3.02] | <.001 | 2.31 (0.37) | | [1.58, 3.03] | <.001 |
| Sex^d^, *_ß01_* | -0.08 (0.10) | [-0.27, 0.10] | .39 | | -0.07 (0.10) | [-0.26, 0.11] | .44 | -0.07 (0.10) | | [-0.26, 0.11] | .44 |
| Age [years], *_ß02_* | -0.01 (0.00) | [-0.02, 0.00] | .051 | | -0.01 (0.00) | [-0.02, 0.00] | .07 | -0.01 (0.00) | | [-0.02, 0.00] | .06 |
| BMI [kg/m^2^], *_ß03_* | 0.02 (0.01) | [-0.01, 0.05] | .24 | | 0.02 (0.01) | [-0.01, 0.05] | .22 | 0.02 (0.01) | | [-0.01, 0.05] | .24 |
| CSE, *_ß10_* | -0.57 (0.02) | [-0.61, -0.52] | <.001 | | -0.57 (0.02) | [-0.61, -0.52] | <.001 | -0.57 (0.02) | | [-0.62, -0.52] | <.001 |
| ilr1, *_ß20_* | 0.11 (0.05) | [0.00, 0.21] | .04 | | -0.02 (0.04) | [-0.10, 0.06] | .57 | -0.10 (0.06) | | [-0.23, 0.02] | .11 |
| ilr2, *_ß30_* | 0.01 (0.05) | [-0.08, 0.10] | .80 | | -0.13 (0.06) | [-0.26, -0.01] | .04 | -0.02 (0.04) | | [-0.10, 0.07] | .70 |
| ilr3, *_ß40_* | 0.08 (0.05) | [-0.01, 0.18] | .09 | | -0.06 (0.04) | [-0.14, 0.03] | .20 | -0.08 (0.04) | | [-0.16, 0.00] | .06 |
| Exercise behavior, *_ß50_* | -0.01 (0.03) | [-0.07, 0.06] | .83 | | -0.01 (0.03) | [-0.08, 0.05] | .72 | -0.01 (0.03) | | [-0.07, 0.05] | .74 |
| **Random effects** ^f^ | SD (VE) | 95% CI | *P* Value | | SD (VE) | 95% CI | *P* Value | SD (VE) | | 95% CI | *P* Value |
| Intercept, *_u0_*  ilr3, *_u1_*  ilr2, *_u1_*  ilr1, *_u1_*  Int.–Slope corr.  Residual, *_r_* | 0.62 (0.39)  0.25 (0.06)  N/A  N/A  -0.28  0.52 (0.27) | [0.55, 0.69]  [ 0.16, 0.35]  N/A  N/A  [-0.59, 0.06]  [ 0.50, 0.54] | <.001  <.001  N/A  N/A | | 0.62 (0.39)  N/A  0.33 (0.11)  N/A  0.17  0.52 (0.27) | [0.55, 0.69]  N/A  [0.19, 0.48]  N/A  [-0.18, 0.50]  [0.50, 0.54] | <.001  N/A  <.001  N/A | 0.62 (0.39)  N/A  N/A  0.34 (0.11)  0.21  0.52 (0.27) | | [0.55, 0.69]  N/A  N/A  [0.20, 0.48]  [-0.14, 0.53]  [ 0.50, 0.54] | <.001  N/A  N/A  <.001 |

^a^Hypothesis 1: ilr1(SB/ (LPA*MVPA*Sleep)), ilr2 (LPA/ (MVPA*Sleep)), ilr3 (MVPA/ Sleep)
^b^Hypothesis 2: ilr1(LPA/ (Sleep *MVPA*SB)), ilr2 (Sleep / (MVPA*SB)), ilr3 (MVPA/ SB)
^c^Hypothesis 3: ilr1(Sleep/ (MVPA*LPA*SB)), ilr2 (MVPA/ (LPA*SB)), ilr3 (LPA/ SB)

^d^compared to female

^e^Unstandardized estimates and standard errors

Abbreviations: BMI = body mass index; CI = Confidence interval; CSE = coping-self-efficacy; ilr = isometric log-ratio coordinate; N/A = not applicable

**Table 2** Adapted multilevel models with daily coping self-efficacy as outcome and exercise behavior as additional fixed predictor

|  | | | H5^a^ | | | | | H6^b^ | | | | | | | H7^c^ | | | | | | | | |
| --- | --- | --- | --- | --- | --- | --- | --- | --- | --- | --- | --- | --- | --- | --- | --- | --- | --- | --- | --- | --- | --- | --- | --- |
|  | | b (SE)^e^ | | | 95% CI | | *P* value | | | b (SE)^e^ | | 95% CI | | *P* value | | | b (SE)^e^ | | 95% CI | | *P* value | |  |
| **Fixed effects**  Intercept, *_ß00_* | 3.42 (0.41) | | | [2.63, 4.21] | | <.001 | | | 3.45 (0.41) | | [2.65, 4.24] | | <.001 | | | 3.44 (0.41) | | [2.65, 4.23] | | <.001 | |  |  |
| Sex^d^, *_ß01_* | 0.16 (0.11) | | | [-0.04, 0.37] | | .13 | | | 0.16 (0.11) | | [-0.04, 0.37] | | .12 | | | 0.17 (0.10) | | [-0.04, 0.37] | | .11 | |  |  |
| Age [years], *_ß02_* | 0.01 (0.01) | | | [ 0.00, 0.02] | | .10 | | | 0.01 (0.01) | | [ 0.00, 0.02] | | .11 | | | 0.01 (0.01) | | [0.00, 0.02] | | .11 | |  |  |
| BMI [kg/m^2^], *_ß03_* | -0.01 (0.02) | | | [-0.04, 0.03] | | .75 | | | -0.01 (0.02) | | [-0.04, 0.03] | | .74 | | | -0.01 (0.02) | | [-0.04, 0.03] | | .74 | |  |  |
| ilr1, *_ß20_* | -0.02 (0.05) | | | [-0.12, 0.09] | | .75 | | | 0.11 (0.05) | | [0.01, 0.21] | | .03 | | | -0.07 (0.05) | | [-0.18, 0.03] | | .17 | |  |  |
| ilr2, *_ß30_* | 0.09 (0.05) | | | [0.00, 0.18] | | .053 | | | -0.03 (0.05) | | [-0.14, 0.07] | | .56 | | | -0.03 (0.04) | | [-0.12, 0.05] | | .45 | |  |  |
| ilr3, *_ß40_* | 0.06 (0.04) | | | [-0.02, 0.14] | | .17 | | | 0.01 (0.05) | | [-0.07, 0.10] | | .76 | | | 0.08 (0.05) | | [-0.02, 0.19] | | .12 | |  |  |
| Exercise behavior, *_ß50_* | -0.01 (0.03) | | | [-0.07, 0.06] | | .86 | | | -0.01 (0.03) | | [-0.08, 0.06] | | .72 | | | -0.01 (0.03) | | [-0.07, 0.06] | | .83 | |  |  |
| **Random effects** | SD (VE) | | | 95% CI | | *P* value | | | SD (VE) | | 95% CI | | *P* value | | | SD (VE) | | 95% CI | | *P* value | |  |  |
| Intercept, *_u0_*  ilr1, *_u1_*  ilr3, *_u1_*  Int.–Slope corr.  Residual, *_r_* | 0.68 (0.47)  N/A  N/A  N/A  0.55 (0.30) | | | [0.61, 0.76]  N/A  N/A  N/A  [0.53, 0.57] | | <.001  N/A  N/A | | | 0.68 (0.47)  0.31 (0.10)  N/A  -0.17  0.54 (0.29) | | [0.61, 0.76]  [0.15, 0.45]  N/A  [-0.57, 0.16]  [0.52, 0.56] | | <.001  .005  N/A | | | 0.68 (0.47)  N/A  0.33 (0.11)  -0.18  0.54 (0.29) | | [0.61, 0.76]  N/A  [0.17, 0.48]  [-0.54, 0.13]  [0.52, 0.56] | | <.001  N/A  .003 | |  |  |

^a^Hypothesis 5: ilr1(SB/ (LPA*MVPA*Sleep)), ilr2 (LPA/ (MVPA*Sleep)), ilr3 (MVPA/ Sleep)
^b^Hypothesis 6: ilr1(LPA/ (Sleep *MVPA*SB)), ilr2 (Sleep / (MVPA*SB)), ilr3 (MVPA/ SB)
^c^Hypothesis 7: ilr1(Sleep/ (MVPA*LPA*SB)), ilr2 (MVPA/ (LPA*SB)), ilr3 (LPA/ SB)

^d^compared to female

^e^Unstandardized estimates and standard errors.

Abbreviations: BMI = body mass index; CI = Confidence interval; ilr = isometric log-ratio coordinate; N/A = not applicable

**Table 3** Adapted multilevel models with daily self-perceived stress and coping self-efficacy as outcome and sleep quality as additional fixed predictor

|  | **Self-perceived stress**  H4^a^ | | | **Coping Self-Efficacy**  H8^a^ | | | |
| --- | --- | --- | --- | --- | --- | --- | --- |
|  | b (SE)^c^ | 95% CI | *P* value | | b (SE)^c^ | 95% CI | *P* value |
| **Fixed effects**  Intercept, *_ß00_* | 2.34 (0.36) | [1.64, 3.04] | <.001 | | 3.29 (0.39) | [2.52, 4.06] | <.001 |
| Sex^b^, *_ß01_* | -0.08 (0.09) | [-0.26, 0.10] | .37 | | 0.17 (0.10) | [-0.03, 0.36] | .11 |
| Age [years], *_ß02_* | -0.01 (0.00) | [-0.02, 0.00] | .04 | | 0.01 (0.00) | [ 0.00, 0.02] | .02 |
| BMI (kg/m^2^), *_ß03_* | 0.02 (0.01) | [-0.01, 0.04] | .29 | | -0.00 (0.02) | [-0.03, 0.03] | .80 |
| CSE, *_ß10_* | -0.53 (0.02) | [-0.57, -0.48] | <.001 | | N/A | N/A | N/A |
| ilr1, *_ß20_* | 0.03 (0.03) | [-0.03, 0.10] | .34 | | 0.04 (0.03) | [-0.03, 0.10] | .28 |
| ilr2, *_ß30_* | -0.00 (0.04) | [-0.08, 0.07] | .90 | | 0.09 (0.04) | [0.02, 0.16] | .01 |
| ilr3, *_ß40_* | -0.13 (0.05) | [-0.24, -0.02] | .02 | | -0.08 (0.05) | [-0.19, 0.02] | .12 |
| Sleep quality, *_ß50_* | -0.00 (0.00) | [0.00, 0.00] | .47 | | 0.00 (0.00) | [ 0.00, 0.01] | <.001 |
| **Random effects** | SD (VE) | 95% CI | *P* value | | SD (VE) | 95% CI | *P* value |
| Intercept, *_u0_*  Residual, *_r_* | 0.61 (0.38)  0.54 (0.29) | [0.55, 0.68]  [0.52, 0.55] | <.001 | | 0.68 (0.46)  0.54 (0.29) | [0.60, 0.75]  [0.52, 0.55] | <.001 |

^a^Hypothesis 4 & 8: ilr1 (MVPA/ (LPA*Sleep*SB)), ilr2 (LPA/ (Sleep*SB)), ilr3 (Sleep/ SB)

^b^compared to female

^c^Unstandardized estimates and standard errors.

Abbreviations: BMI = body mass index; CI = Confidence interval; CSE = coping-self-efficacy; ilr = isometric log-ratio coordinate; N/A = not applicable
